# T-Cell Immunodeficiencies With Congenital Alterations of Thymic Development: Genes Implicated and Differential Immunological and Clinical Features

**DOI:** 10.3389/fimmu.2020.01837

**Published:** 2020-08-14

**Authors:** Giuliana Giardino, Carla Borzacchiello, Martina De Luca, Roberta Romano, Rosaria Prencipe, Emilia Cirillo, Claudio Pignata

**Affiliations:** Department of Translational Medical Sciences, Pediatric Section, Federico II University of Naples, Naples, Italy

**Keywords:** Thymus, *FOXN1* gene, *PAX1* gene, Pax 1/9, CHARGE, *CHD7* gene, *TBX1* gene, DiGeorge anomaly

## Abstract

Combined Immunodeficiencies (CID) are rare congenital disorders characterized by defective T-cell development that may be associated with B- and NK-cell deficiency. They are usually due to alterations in genes expressed in hematopoietic precursors but in few cases, they are caused by impaired thymic development. Athymia was classically associated with DiGeorge Syndrome due to *TBX1* gene haploinsufficiency. Other genes, implicated in thymic organogenesis include *FOXN1*, associated with Nude SCID syndrome, *PAX1*, associated with Otofaciocervical Syndrome type 2, and *CHD7*, one of the genes implicated in CHARGE syndrome. More recently, chromosome 2p11.2 microdeletion, causing *FOXI3* haploinsufficiency, has been identified in 5 families with impaired thymus development. In this review, we will summarize the main genetic, clinical, and immunological features related to the abovementioned gene mutations. We will also focus on different therapeutic approaches to treat SCID in these patients.

## Introduction

The thymus is a primary lymphoid organ which plays a pivotal role in the development of mature T cells from immature bone marrow CD34+ precursors. Together with the parathyroid glands, it develops from the 3 pharyngeal pouch (PP) (1). The epithelial components of the thymus derive from the endothelial layer, while the mesenchymal capsule derives from neural crest, originated from ectoderma (1, 2). The first stage of thymic development is independent of the transcription factor forkhead box N1 (*Foxn1*) expression (3). In this phase, Paired box 1 (*Pax1*), Eyes absent homolog 1 (*Eya1*), sine oculis homeobox (*Six*), homeobox A3 (*Hoxa3*), and T-box 1 (*Tbx1*) drive the outgrowth of the thymic epithelial anlage from the 3rd PP (1, 4, 5). *Hoxa3* and *Eya1* are also implicated in the development of neural crest derived mesenchymal cells (6–8). Studies suggest that chromodomain helicase DNA-binding 7 (*Chd7*) might be implicated in the development of both neural crest cell-derived mesenchyme and pharyngeal endoderm-derived thymic epithelial cells (TECs). *Pax3* and *Hoxa3* expression in mesenchymal cells allows the detachment of the thymic lobes from the pharynx (9, 10). Thymus development in early and late stages is regulated by the interactions among various cell types. The thymus three-dimensional (3D) architecture allows a proper intercellular cross talk (3). In the first phase of organogenesis, mesenchymal cells release bone morphogenetic protein 4 (Bmp4), Bmp2, fibroblast and insulin growth factors (Fgf, Igf), Wnt proteins, and retinoic acid supporting the differentiation of TECs into cortical (cTECs) and medullary (mTECs) subsets (7, 8, 11–14). In the second phase, Foxn1 induces the expression of chemokine (C-C motif) ligand 25 (*CCL25*), delta like canonical Notch ligand 4 (*Dll4*), and *Hoxa3*, allowing thymocyte recruitment and TECs differentiation in cTECs and mTECs. Mesenchymal cells are implicated in the recruitment of hematopoietic thymic seeding progenitors, as well (15, 16). Thymocytes participate to TECs differentiation process through the release of epidermal growth factor (Egf) and lymphotoxin factors (17–19). In support of this, mice with defective T-cell development show defective organization of the thymic medulla (20, 21), that is restored after stem cell transplantation (21, 22).

**Figure 1:**
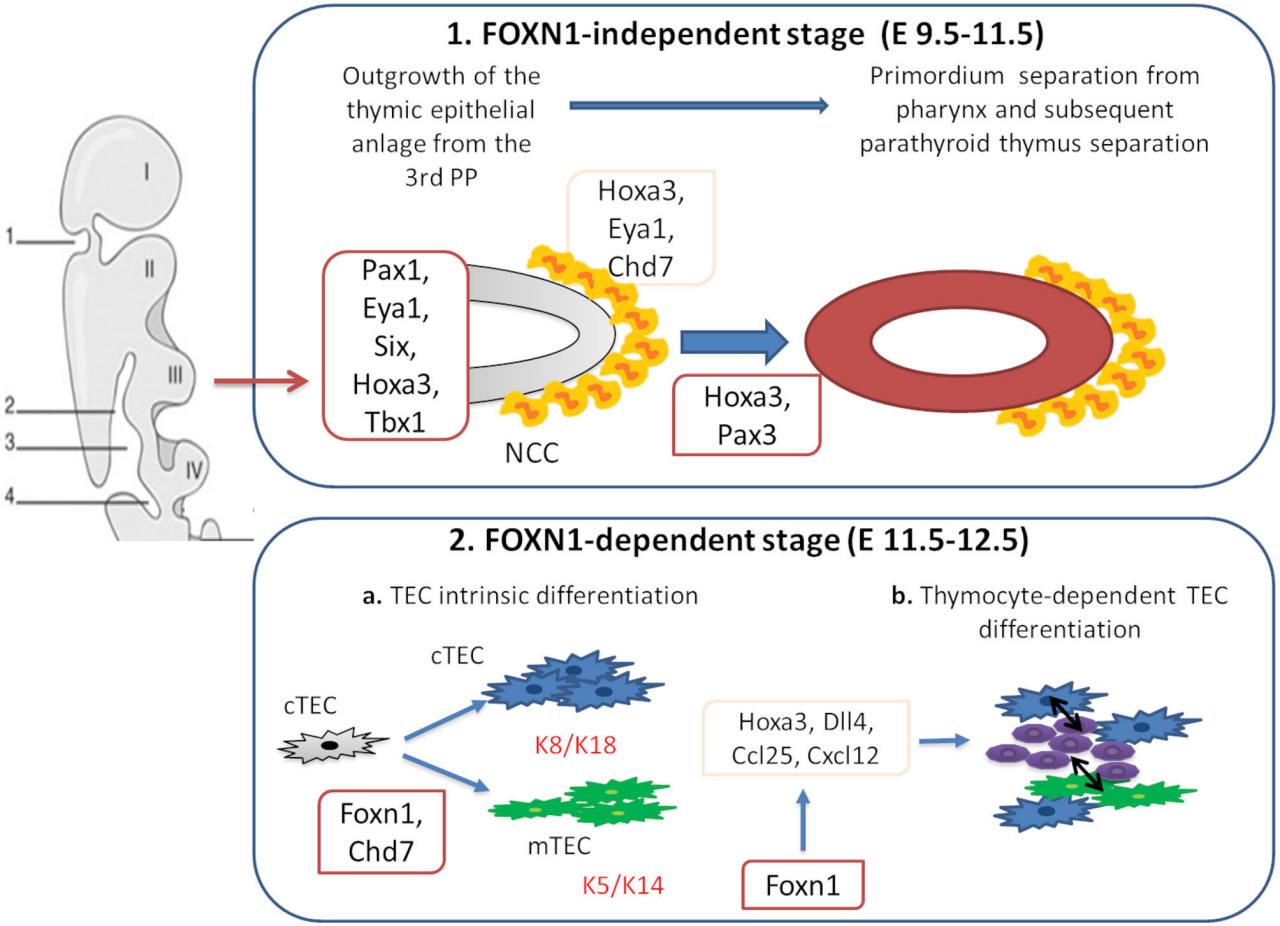
Genes involved in thymus organogenesis. *Eya1, Six, Hoxa3, Tbx1*, and *Chd7* take part to the first stages of thymus development from neural crest cells (NCCs) in the posterior part of the 3rd pharyngeal pouch (PP). This step is independent from *Foxn1* expression. In the second stage, *Foxn1* regulates the expression of *Hoxa3, Dll4, Ccl25*, and *Cxcl12*, necessary for the thymic epithelial cells (TECs) differentiation. During this phase, cTECs (expressing K8 and K18 keratin type) and mTECs (expressing K5 and K14 keratin type) originate from the same bi-potential TECs progenitor. *Chd7* is also critical for the development of cortical and medullary TECs from pharyngeal endoderm. The crosstalk between TECs and developing thymocytes is required to generate mature TECs and functional T cells.

Bone marrow derived hematopoietic stem cells (HSCs) enter the thymus through cortico-medullary junction, where they proliferate (23). The V(D)J rearrangement of the double negative (DN) thymocytes T-cell receptor β (TCRβ) gene takes place in the thymic cortex (24, 25). Membrane expression of pre-TCR complex is necessary for the expression of the co-receptors CD4 and CD8, as well as V-J rearrangement of the TCRα genomic region (26). Double positive thymocytes with a functional TCR-αβ receptor capable of binding to self-MHC ligands are positively selected (27–29). This process is regulated by Prss16 and β5t, which are expressed in cTECs (30–34). Into medulla, self-reactive thymocytes are deleted through the negative selection, a process mediated by dendritic cells and Aire-expressing mTECs (35, 36). Mutations in genes implicated in different steps of thymic development, including *FOXN1, PAX1, TBX1, CDH7* impair T-cell development in humans (Figure 1). Alterations of the immune system in these conditions range from an isolated reduction of T-cell count to severe combined immunodeficiency (SCID). This review is focused on definition of the role of different genes implicated in thymus development and of primary immunodeficiencies (PIDs) due to their deficiency. Moreover, therapeutic options for PIDs with congenital athymia are discussed.

## Gene Function and Related Syndrome

### *FOXN1* Deficiency and Nude-SCID Syndrome

*FOXN1* is located on chromosome 17q11.2 and it is composed of eight exons (30 kb) (37, 38). This gene is a member of the Fox gene family that comprises different Winged helix transcription factors implicated in development, metabolism, cancer, and aging (39). During fetal life, *FOXN1* is expressed in mesenchymal and epithelial cells including liver, lung, intestine, kidney, and urinary tract. In postnatal life, its expression is restricted to keratinocytes and TECs (40, 41). FOXN1 is involved in development, function and maintenance of hair follicles, and TECs (Figure 1) (42–44). The expression of *FOXN1* in TECs leads to the production of chemokines, such as C-X-C motif chemokine ligand 12 (CXCL12), implicated in the recruitment of hematopoietic progenitors and of molecules, as DLL4 notch ligand, implicated in maturation of the progenitors toward the T-cell line (Figure 1) (45–47). Studies in mice showed that Foxn1 plays a pivotal role in morphogenesis of the 3D architecture (48, 49) by inhibiting a basic morphogenetic pattern of tubulogenesis and inducing the expression of genes that drive TECs differentiation (50). FOXN1 also regulates *Prss16* and β*5t* gene expression, implicated in positive selection (45, 51). Knockout of *Hoxa1, Pax1, Pax9, Eya1*, or *Six1* affects *Foxn1* expression resulting in impaired thymic function (52). *Foxn1* expression is also regulated by signals from the surrounding endothelium and mesenchyme, including Bmp4 and Wnt (53, 54).

In epidermis, FOXN1 is expressed in keratinocytes of suprabasal layer, where these cells stop proliferating and start the terminal differentiation (55). In keratinocytes, FOXN1 regulates the transcription of more than 50 genes, including protein kinase B and C (PKB and PKC). PKC is an inhibitor of human hair follicle growth *in vitro* (56–58). It is up-regulated in Foxn1–/– mice while its activity is suppressed in mice overexpressing Foxn1 gene in which differentiation is inhibited. Studies conducted on human keratinocytes have confirmed the role of the gene in the differentiation of the epidermis (58).

Homozyous *FOXN1* mutations cause Nude SCID syndrome, first observed in two Italian sisters presenting with congenital universal alopecia, nail dystrophy, and severe T-cell immunodeficiency with rudimentary thymus (59). In the last few years, neonatal screening and next generation sequencing techniques have led to the identification of subjects with novel homozygous, compound heterozygous, and heterozygous mutations (60). Homozygous patients suffer from immunodeficiency with susceptibility to pneumonia, chronic diarrhea, candidiasis or mycobacterial infections, and Omenn syndrome (59, 61–64). Most of the heterozygous patients show nail dystrophy, usually presenting as leukonychia and minor immunological changes (60, 65, 66). Recurrent infections and atopic dermatitis are observed only in a minority of the patients (60).

Immunological features in patients with homozygous *FOXN1* mutations so far reported include reduction of T lymphocytes, particularly CD4+ T cells (61–64), reduction of T-cell receptor excision circles (TRECs) and naive T CD45RA+ lymphocytes, with increase of T memory CD45RO+ lymphocytes (62–64). In addition, an increase in the DN CD4-CD8- lymphocytes in the periphery (61–63), a reduction in CD31+ cells, recently emigrated from the thymus was observed in few cases (63). Proliferative response to phytohemagglutinin (PHA) is usually poor or absent and TCR repertoire is oligoclonal (59, 61–63). Although natural killer (NK) and B cells are usually numerically normal, they may be functionally compromised, with impaired production of specific antibodies. Recent studies proved that *FOXN1* haploinsufficiency, caused by *FOXN1* heterozygous mutations, may be associated with T cell-lymphopenia in infants showing low TRECs at newborn screening (60). A progressive normalization of CD4 count is observed in adulthood in heterozygous subjects while CD8 are usually persistently low (60). The increase in CD4 levels is associated to persistently low CD45RA levels suggesting a mechanism of homeostatic proliferation. A more significant homeostatic expansion in CD4 than CD8 might explain the difference in CD4 and CD8 T cells. Alternatively, another hypothesis is that *FOXN1* dosage plays a stricter role in the expression of CD8 development genes (60).

## *PAX1* Deficiency and Otofaciocervical Syndrome Type 2

*PAX1* is a member of a family of genes that encodes transcription factors implicated in embryogenesis in vertebrates (67). It is located on chromosome 20p11.22 and contains 5 exons (10 Kb) (67, 68). This gene is expressed in fetal mesenchymal cells in the body of intervertebral disks and plays an important role in formation of the segmented vertebral column in humans (69). In mice, it is also expressed in cochlea and has a role in hearing process (70). *PAX1* is also implicated in thymus organogenesis by contributing to the outgrowth of the thymic epithelial anlage from the 3rd PP and to the regulation of TECs differentiation/survival balance (Figure 1) (71, 72). During pre-natal life, it is expressed in the 3rd PP from E9.5, while in the post-natal thymus it is only expressed in cTECs (72) (Figure 1). In mice, *Pax1* deficiency is associated with moderate thymic hypoplasia (72) that is more severe only when it is associated with *Hoxa3* haploinsufficiency (73). On the contrary, in humans, *PAX1* deficiency is associated with severe thymic hypoplasia, leading to SCID (74). The difference between human and mouse phenotype may be due to a compensatory contribution by murine *Pax9* when *Pax1* is lacking (75).

Otofaciocervical syndrome (OTFCS) is an autosomal dominant disorder characterized by short stature, facial dysmorphism (long face, narrow mandible), shoulder girdle abnormalities, hearing loss, and mild intellectual disability (76). Two different forms of OTFCS have been described but thymus development is only affected in OTFCS2, caused by *PAX1* mutations. Different biallelic deleterious *PAX1* variants cause OTFCS2 and SCID, characterized by absent thymic shadow, chronic diarrhea, recurrent respiratory infections, pneumonia, and also Omenn Syndrome (74). Different severity of OTFCS2 phenotype is described in different families (70, 71, 77).

The immunological phenotype of patients with OTFC2 was described very recently in six patients who showed T-cell lymphopenia, impaired proliferative response to mitogens, and normal levels of B and NK cells. In one of them, 98% of CD4 and CD8 T cells were CD45RO+ cells, while CD45RA+CD31+ cells and TRECs were undetectable. TCRVβ analysis showed an oligoclonal spectrum. Lymph node biopsy of another patient showed absence of germinal centers and almost total absence of CD3+ T cells. Patients with Omenn Syndrome also showed eosinophilia and increased IgE levels (74).

## Digeorge Syndrome and 22q11.2 Deletion

DiGeorge syndrome (DGS) is usually associated with 3 or a 1.5 Mb *de novo* microdeletion of 22q11.2 (78, 79). In the central region of deletion maps *TBX1* gene containing 9 exons (80) and encodes a Tbx transcription factor, implicated in the regulation of nearly 2,000 genes (81, 82). It is strongly expressed in the 3rd and 4th PP endoderm and in the 4th pharyngeal arch arteries (83, 84), in the otic vesicle, vertebral column, later in tooth bud and, at a lower extent, in the brain (85). It is implicated in pharyngeal arch segmentation and outgrowth of the TECs from the 3rd PP (Figure 1). However, it should be noted that Tbx1 is not expressed in the thymic anlage and thus it is not directly implicated in TECs development (86). On the contrary, Tbx1 enforced expression within the 3rd PP represses TECs development (86). Reduced levels of Tbx1 in 22q11.2 deletion syndrome (22q11.2DS) impair the development of neural crest-derived mesenchymal cells that surround the 3rd PP, leading to thymic hypoplasia (87). TBX1 regulates the expression of secreted Fgfs molecules, namely Fgf8 and Fgf10, implicated in the control of TECs proliferation, differentiation, migration, and survival (88). Ffg receptor IIIb (FgfR2-IIIb) regulates the cascade of *Hox3* paralogs transcription factors, *Pax1/Pax9*, and winged helix nude (*Whn*) (Figure 1) (89). Moreover, TBX1 interferes with the ability of small mother against decapentaplegic 1 (SMAD1) to bind SMAD4, preventing effective Bmp4 signaling (82). Bmp4 also contributes to early thymus and parathyroid morphogenesis (90). Isolated *TBX1* mutations may be rarely reported in patients with DGS (91) and *TBX1* gain-of-function mutations can result in the same phenotypic spectrum of loss-of-function mutations (92).

An embryonic phenocopy of DGS with impaired thymus development can be observed because of the lack of retinoic acid during gestation (79, 93, 94). Retinoic acid is able to regulate the expression of *Tbx1* and other molecules implicated in thymus organogenesis including *Pax1, Pax9, Hoxa3, Fgf8*, and *Bmp4* (95). Other epigenetic factors, including maternal diabetes (93) or prenatal exposure to retinoic acid or alcohol may also explain the alteration of thymus development in DGS (96–101). Recently, we reported on a 7-year-old DGS patient born to a mother with gestational diabetes mellitus in whom a 371 Kb-interstitial deletion of 3p12.3, involving the Zinc Finger Protein 717 (*ZNF717*), MicroRNA-1243 (*MIR-1243*), and *MIR-4273* genes was identified (102). MiRNAs are small, non-coding RNAs involved in the modulation of gene expression by targeting messenger RNAs for degradation, translational repression, or both (103). miRNA-4273 regulates the expression of Bmp3, a member of the transforming growth factor β superfamily, involved in thymus and kidney development (104). The expression of other miRNAs, including MIR-185, and MIR-150 can be impaired in 22q11.2DS patients (105). MIR-185 reduction increases Bruton's tyrosine kinase (Btk) expression leading to autoantibody production while MIR-185 increase leads to dose-dependent T-cell lymphopenia (105, 106). Reduced MIR-150 expression contributes to the reduction of T and B cells (107). Dysregulation of miRNA biogenesis, due to DiGeorge Critical Region Gene 8 (*DGCR8*) haploinsufficiency, is implicated in the pathogenesis of immunological, cardiac, endocrinological, and neurological phenotype (105, 108). Other genes implicated in the immune response are included in the deleted region. Alterations of CrK-like (*CRKL*), a gene encoding a 39-kDa adapter protein belonging to the Crk family, implicated in many cellular functions, including cell migration and adhesion, are associated with impaired T-cell proliferation in response to TCR triggering (109).

DGS has a prevalence of 1:4,000 newborns (79, 110). Most of these patients present with thymic and parathyroid hypoplasia, congenital heart defects, and craniofacial dysmorphisms (78, 79). Thymic development ranges from athymia in complete DGS (cDGS) to a completely normal thymus development in partial DGS (pDGS), resulting in a variable spectrum of T-cell deficiency (78, 79, 111). cDGS is reported in about 1.5% of the patients (111). Patients with DGS show a wide spectrum of T-cell alterations ranging from completely normal T-cell development to cDGS with absent thymic development (112, 113). In 22q11.2DS patients, thymus is usually small or hypoplastic. The size of the thymus does not predict the levels circulating T cells. In fact, microscopic rests of TECs may be present at aberrant locations (114). Low CD3+ T-cell percentage is the most common T-cell defect, followed by low CD3 number (78). CD4 and CD8 compartments are similarly affected (78). T-cell number and percentage tend to increase during the follow up starting from the first year of life (78). Naive CD4 and CD8 lymphocytes are lower in pDGS patients compared to controls independently of age and they decline more rapidly with age (78). The improvement of the lymphopenia with age is not due to a recovery of the thymic function but to the peripheral homeostatic expansion of the available T cells, as suggested by the evidence that T cells are predominantly or almost exclusively of a memory phenotype, TRECs are low and the repertoire is oligoclonal (78, 115, 116). T-cell proliferation, total immunoglobulins, and specific antibody response to vaccines are typically normal. IgM levels are often low, and some patients may show selective IgA deficiency (78). The study of the thymic architecture and thymocyte development in thymi obtained from pediatric pDGS patients revealed a reduction of mature CD4+ and CD8+ T cell frequency, associated with reduced proportion and function of T regulatory cells (Tregs) (117).

The majority of DGS patients suffer from chronic otitis media, which correlates with primarily conductive hearing impairment (78). Most patients have increased susceptibility to mild infections and only rarely to atypical or severe infections (78). Abnormalities of PP derivatives, predisposing to bacterial colonization, more than immune defects are implicated in this susceptibility (78). Autoimmune diseases, mainly presenting as rheumatoid diseases and idiopathic thrombocytopenia purpura, are reported in ~10% of DGS patients (113, 116, 118). Predisposition to autoimmunity in DGS patients is partially explained by the mechanism of lymphopenia induced T-cell homeostatic proliferation together with the reduction of natural Tregs (nTregs) (117, 119).

## *CHD7* Haploinsufficiency and Charge Syndrome

CHARGE (coloboma, heart defects, atresia choanae, growth retardation, genital abnormalities, and ear abnormalities) syndrome is associated to haploinsufficiency in *CHD7* gene, located on chromosome 8q12 (120). CHD7 is implicated in chromatin organization of mesenchymal cells, derived from the cephalic neural crest (121). Impaired *CHD7* expression correlates with defects in neural crest cells, cephalic mesenchyme, pharyngeal arches, brain, otic vesicle and, in the mesoderm of the developing heart, especially in the outflow tract of the heart (122, 123). CHD7 is critical for the development of cTECs and mTECs from pharyngeal endoderm by regulating *Bmp4*, which in turn regulate *Foxn1* expression (Figure 1) (124). *Chd7* deficiency is also associated with down-regulation of *Ikaros*, Interelukin 7 receptor (*Il7r*), recombinase activating gene 1 (*Rag1*) (124). Studies suggest that *CHD7* can also regulate *TBX1* expression (74, 125).

CHARGE syndrome is characterized by different degrees of thymic alterations and even by complete thymic aplasia, resulting in combined immune deficiency (126). A wide spectrum of T-cell deficiency and isolated humoral immune deficiency may be observed in patients with CHARGE syndrome (126). They may show severe T-cell deficiency resembling SCID or, similarly to DGS, they may present transient lymphopenia, that usually normalizes over time. A close association between lymphopenia and hypocalcemia has been identified (115). Severity of the T-cell lymphopenia relates to the degree of thymic hypoplasia (127). CHARGE patients also show decreased naive CD4 and CD8 T-cells, peripheral T cells, and TRECs. Peripheral B-cell differentiation and immunoglobulin production are normal but in a 3rd of the patients an abnormal expression of IgM on class-switched memory B cells and diminished production of specific antibodies may be observed (127). Selective antibody deficiency tends to resolve spontaneously over time (127). In a recent paper, immunological features were compared between CHARGE and DGS patients. Total lymphocyte count was slightly lower in DGS patients compared to CHARGE patients and persistent lymphopenia was more common in DGS patients than in CHARGE. IgM levels were significantly lower in DGS patients compared to CHARGE (128).

Most of the patients have increased risk of recurrent infections, including recurrent otitis media, sinusitis, conjunctivitis, dermatitis, respiratory tract infections, pneumonia, and sepsis (127). Severe or atypical infections may also be reported including recurrent oral candidiasis, recurrent severe infections, septic shock, and chronic viral infections (129). As for partial DGS also in CHARGE syndrome the frequent need of invasive operative procedures and anatomical alterations, including extensive ear, sinus, nasal and palatal malformations, altered Eustachian tube anatomy, gastroesophageal reflux, or neurological abnormalities with compromised drainage and aspiration may help explain the susceptibility to infections (127). Immune defects including impaired thymus development and subsequent humoral deficiency may also contribute to the susceptibility to infections but at a lower extent (128). Patients with CHARGE syndrome also show increased risk of atopy, reported in 65% of the patients, usually presenting as food allergy (128). Increased susceptibility to autoimmune disorders may also feature this syndrome (129). Patients with complete athymia may present with atypical SCID (Omenn-like) phenotype (130).

## 2p11.2 Microdeletion and *FOXI3* Haploinsufficiency

A microdeletion at 2p11.2 has been identified in five families presenting with DGS features, including hypocalcaemia, asymmetric crying face, low TRECs and T-cell lymphopenia, without typical facial dysmorphism, and heart abnormalities. *FOXI3*, a member of FOX family transcription factors, implicated in development of brachial arch-derived structures was considered the candidate gene for this phenotype (131). Early in embryonic development *Foxi3* is broadly expressed in the pre-placodal ectoderm surrounding the neural plate, from which all craniofacial sensory organs derive (132, 133). Subsequently, its expression is restricted to the region from which otic and epibranchial placodes derive and finally to the ectoderm and endoderm of the pharyngeal arches. A deletion of *FOXI3* gene has been recently identified in a patient with left congenital aural atresia and ipsilateral internal carotid artery agenesis (134). *Foxi3* is also implicated in the differentiation of the epithelial cells within the epidermis as suggested by the identification of *Foxi3* heterozygous mutations in several hairless dog breeds with hair follicle and teeth defects (135) and in thymic cortico-medullary differentiation. *Foxi3* is implicated in segmentation of the pharyngeal apparatus and LOF of *Foxi3* alone or in combination with *Tbx1* LOF, leads to failure of the pharyngeal arch segmentation due to the inability of the epithelia to properly invaginate with subsequent thymic hypoplasia/aplasia (136).

The main clinical features of the different syndromes are compared in Table 1.

**Table 1:** Comparison of the main clinical features among different congenital disorders of thymic development.

	**DGS**	**FOXN1**	**OTFC2 syndrome**	**CHARGE syndrome**
Dysmorphic features	Low ears, telecanthus, down/up slanting palpebral fissures, short philtrum, velopharingeal insufficiency	Epicanthal folds	Ear malformations, preauricular fistulas, vertebral malformations, lacrimal ducts abnormalities, abnormal clavicles and scapulae, retrognathia, downslanting palpebral fissures, long eyelashes, blue sclerae, epicanthal folds, small nose	Ear abnomalities, coloboma, choanal atresia, cleft palate
Cutaneous alterations	–	Alopecia, nail distrophy	–	–
Thymic alterations	Aplasia (cDGS), hypoplasia/normal (pDGS)	Aplasia (homozygous mutations), hypoplasia (heterozygous mutations)	Aplasia/hypoplasia	Aplasia/hypoplasia
Cardiopathy	Tetralogy of fallot, ventricular septal defect, type B interrupted aortic arch, truncus arteriosus, right aortic arch, aberrant right subclavian artery	–	–	Atrial septal defects, ventricular septal defect, patent ductus arteriosus
Infections	Recurrent/severe infections (cDGS) recurrent infections (pDGS)	Recurrent/severe infections (homozygous mutations), recurrent infections (heterozygous mutations)	Recurrent/severe infections	Recurrent/severe infections
Omenn syndrome	+ (cDGS)	+ (homozygous mutations)	+	+

*DGS, DiGeorge syndrome; cDGS, complete DGS; pDGS, partial DGS; OTFC2 syndrome, Otofaciocervical syndrome type 2; CHARGE syndrome, coloboma, heart defects, atresia choanae, growth retardation, genital abnormalities, and ear abnormalities syndrome*.

## Treatment Options for Athymic Conditions

Hematopoietic stem cell transplantation (HSCT) represents the cornerstone for the treatment of SCID. However, in SCID due to genetic defects that impair development and function of the thymic epithelium, theoretically thymus transplantation would represent the most appropriate therapy. Thymic tissue is obtained from infants undergoing to median sternotomy for open heart surgery. Cultured postnatal human thymic tissue is then transplanted in thin slices into the quadriceps muscle (137). In cases with successful transplant, few months after the transplant the graft is colonized by host stem cells and is able to support normal thymopoiesis (138) leading to the development of mature naive T-cell with diverse TCRVβ repertoire and able to proliferate in response to mitogens. T-cell levels in surviving patients are usually low for age but are sufficient to respond to viral, disseminated, and other infections leading to a resolution of the immunodeficiency (139). In some cases, reduced thymic output may be explained by other comorbidities, such as heart failure and hypoxia stress, that may cause hypoperfusion of the graft (139). The success of the transplant may be also limited in patients with active viral infections since the virus itself, its treatment, or both might inhibit the thymopoiesis (139). In patients with successful transplant, naive T-cells are usually detected within 6 months after the transplant (139, 140). Antibody responses and immunoglobulin levels normalize (62) even though numbers of class-switched memory B cells may remain relatively low. The success of the transplant is not correlated with the amount of tissue transplanted, HLA matching, culture conditions or immunosuppression of the recipient (141). Immunosuppression can be used to delete reactive oligoclonal T cells and mature T cells responsible of graft-versus-host disease and graft rejection (137, 139). Overall survival in DGS is 75% and mortality is usually related to pre-transplantation morbidity, mainly viral infections, and chronic lung disease (115, 140, 142). Thymus transplantation has been recently used to treat 2 patients with *FOXN1* deficiency and both survived (62, 143, 144) while its use in athymic CHARGE and OTFC2 patients has never been reported. Autoimmune disorders, including thyroiditis and severe cytopenia, represent the most common complication after thymus transplant (139, 140).

Adoptive transfer of mature T cells from human leukocyte antigen identical siblings through bone marrow transplantation represents an alternative to thymus transplant to treat SCID in athymic patients (145). However, only post-thymic T cells engraft in this case and naive T cells do not develop. Survival after matched unrelated donor and matched sibling transplantations in cDGS were reported as being 33 and 60%, respectively (145) while in CHARGE out of six patients treated with HSCT three had graft vs. host disease and three died post-transplant (125, 130, 146, 147). Long-lasting survival patients after matched sibling donor transplantation are reported (148). Four *FOXN1* deficient patients underwent HSCT and two survived (59, 63, 64, 149). One of them is currently well at 22 years of age (unpublished data). The study of the T cell compartment in this patient, 5 years after HSCT showed a marked reduction of CD4CD45Ra levels with normal CD8CD45Ra levels. However, TCRVβ repertoire, was largely impaired in the CD8 subset (150). Six patients with OTFC2 were treated with allogeneic HSCT. T-cell reconstitution was not observed in any of the patients, despite successful engraftment in three of them. In one of the cases with successful engraftment all the T cells showed a memory (CD45R0+) phenotype, but no *de novo* generation of a polyclonal repertoire of naive T cells was observed. The remaining 2 patients showed persistent T cell lymphopenia leading to severe and recurrent infections, and death for septic shock in one patient and to severe autoimmune hemolytic anemia in the other (74). Together, these data indicate that HSCT may be unable to correct the profound T cell immunodeficiency of this disease.

## Conclusions

In conclusion, in this review we described different pathways involved in thymus development and the clinical phenotypes associated with their impairment. We also summarized the outcome related to different therapeutic approaches to these disorders. We highlighted the clinical importance of the early detection of a defect in the pathways involved in T-cell development. With the recent introduction of newborn screening programs a timely identification of patients affected with defects of the T-cell development before the onset of the symptoms is now possible, and this prompted the definitive treatment with HSCT. This has been proven effective in improving the prognosis. However, in some cases HSCT is not required for the management of infants with T-cell lymphopenia at birth, since the T-cell development tend to improve with age (prematurity, FOXN1 haploinsufficiency). In other cases, HSCT may not be curative since the defect involves thymus development. However, genome-wide association studies have shown that a large proportion of variants likely to cause human disease are located outside of the protein-coding domains, so whole genome wide approaches might lead to proper identification and thus correct treatment plans for immunodeficiency disorders resulting from aberrant expression of some of the genes discussed in the present review.

## Author Contributions

All authors listed have made a substantial, direct and intellectual contribution to the work, and approved it for publication. GG, CB, MD, EC, and RR reviewed the literature, organized and wrote the manuscript. CP wrote the manuscript and supervised all the work. RP did the picture. All authors reviewed and approved the manuscript.

## Conflict of Interest

The authors declare that the research was conducted in the absence of any commercial or financial relationships that could be construed as a potential conflict of interest.
